# Against the Epistemological Primacy of the Hardware: The Brain from Inside Out, Turned Upside Down

**DOI:** 10.1523/ENEURO.0215-20.2020

**Published:** 2020-08-07

**Authors:** David Poeppel, Federico Adolfi

**Affiliations:** 1 Max Planck Institute for Empirical Aesthetics, Frankfurt 60322, Germany; 2 Department of Psychology, New York University, New York, NY 10003; 3 Max-Planck-New York University Center for Language, Music, and Emotion (CLaME), New York, NY 10003

## Abstract

Before he wrote the recent book *The Brain from Inside Out*, the neuroscientist György Buzsáki previewed some of the arguments in a paper written 20 years ago (“The brain-cognitive behavior problem: a retrospective”), now finally published. The principal focus of the paper is the relationship between neuroscience and psychology. The direction in which that research had proceeded, and continues now, is, in his view, fundamentally misguided. Building on the critique, Buzsáki presents arguments for an “inside-out” approach, wherein the study of neurobiological objects has primacy over using psychological concepts to study the brain, and should, in fact, give rise to them. We argue that he is too pessimistic, and actually not quite right, about how the relation between cognition and neuroscience can be studied. Second, we are not in agreement with the normative recommendation of how to proceed: a predominantly brain first, implementation-driven research agenda. Finally, we raise concerns about the philosophical underpinning of the research program he advances. Buzsáki’s perspective merits careful examination, and we suggest that it can be linked in a productive way to ongoing research, aligning his inside-out approach with current work that yields convincing accounts of mind and brain.

## Introducing the Problem and the Players

One of the foundational aims of neuroscience is to understand behavior, in the broadest sense. To be sure, not all of neuroscience is concerned with perception, attention, memory, emotion, language, action, and all the other fascinating features that form the basis of our experience. A substantial portion of the approaches that comprise the neurosciences are dedicated to understanding in as much detail as possible the biological and physiological infrastructure of the brain. But to investigate, debate, and speculate about how the brain forms the basis for the mind is deeply important, exciting, and of course great fun. A colleague who has worked for a long time at this intersection is György Buzsáki (henceforth GB), who, building on his enormous experience with neurobiological experimentation, has turned every so often to take stock of the field and its larger issues. GB’s reflections are expressed in a particularly provocative manner in an older paper and a newer book. The 20-year-old paper (“The brain–cognitive behavior problem: a retrospective”), in which GB pointed to some critical shortcomings at the intersection of brain and behavior, sets the stage for his recent book (*The Brain from Inside Out*), in which he further develops a lot of the early threads (as well as ideas articulated in his previous book, Buzsáki, 2006), and advances arguments about how to investigate thorny questions at the nexus of brain and mind.

Here, we respond to some of the positions that are taken in the old article (Buzsáki, 2020) and expanded in the new book (Buzsáki, 2019). Before we get into nitty-gritty argumentation, a few points are in order, for context. First, it is really important that scientists like GB zoom out and think about what is going on in the field. The vast majority of neuroscientists never engage with the more philosophically motivated questions, we think at their peril. It is laudable that GB regularly investigates the assumptions of what we do and provides high-level perspective. We consider this a critical service to the field, whether or not one agrees with the positions that are articulated. Second, unsurprisingly, there are many, many points of agreement between us. But to write a paper wherein we, for the most part, simply say how “very much on the right track all of this is” would be unhelpful, and perhaps even (and this would be unforgivable) boring. There is, both in the older paper and in the new book, a huge amount to like! For the sake of pedagogy, though, and to highlight the issues on which we truly disagree, we focus here on the arguments we consider “not quite on the right track,” or to be more blunt, in our view just plain wrong. Third, from the point of view of “meta-scientific disposition,” we occupy a very similar stance as GB… We, too, are deeply skeptical about a lot of the research that attempts to link brain and behavior. In fact, we are arguably crankier and more polemical than GB, who is always thoughtful and polite. In any case, we all agree that there is a tremendous amount of research that is fundamentally misguided, although we may come to that conclusion from slightly different perspectives and with different consequences. Finally, full disclosure, GB is a treasured colleague and friend. It is, in that sense, a special privilege to be allowed to have, in a more public setting, a spirited debate between friends that we all hope leads to progress.

We proceed in three parts. First, we address GB’s criticism that the relationship between brain and behavior has been dominated by the terminology of psychology. This part of the critique is focused on cognitive neuroscience, broadly construed. We suggest that GB is being unnecessarily uncharitable to the psychological and cognitive sciences. Second, we discuss the normative recommendation that he outlines. Here, GB embraces something like the “epistemological primacy of the hardware,” and he outs himself, somewhat surprisingly, as a “radical implementationist.” We consider the position and point to some problematic consequences of this stance. Finally, we raise some points about the philosophical position that GB favors; we question his predisposition and provide some cautionary remarks. It goes without saying that the topics we are arguing about are, for the moment, at the very edge of anyone’s understanding. Our epistemic limitations prevent us from deeper, explanatory accounts. But putting one’s cards on the table, philosophically and methodologically, at least provides some ideas that one can agree or disagree with, in the service of progress.

## Don’t Throw the Cogneuro Baby Out with the Bad-Science Bathwater

The starting point for GB’s reflections is the problematic relationship between the “object” of neuroscience (i.e., the hardware/wetware itself) and the psychological terms that are taken to be the phenomena to be explained. The concerns are motivated by studies in cognitive neuroscience, although the argument is taken to extend to behavioral neuroscience in animal research, as well. There has, indeed, been too much work that is indefensible or just plain silly. The development and easy availability of noninvasive tools, which is an important and fantastic advance in terms of methodology, has not necessarily been accompanied by equally fantastic experimentation. Although there has been impressive progress on measurement technology with respect to the spatial resolution and temporal resolution of the devices, the “conceptual resolution” has not been as impressive. Like GB, we agree that there have been many cases of, at best, hard-to-interpret correlational cognitive neuroscience observations. One regrettable issue is that the ability to pinpoint functional anatomic regions has been taken to be more explanatory than is legitimate. We, too, have been grappling with this issue for a long time. For example, in early imaging studies of language processing, the results pointed in many different directions, with little neurobiological systematicity (Poeppel, 1996). The tendency to interpret localization as explanation has also been problematic in many studies, and certainly caused egregious misinterpretations in the popular press (Poeppel, 2008). But are these shortcomings principled or practical? In our view, GB is legitimately harsh on what is bad research, but he is throwing out the cognitive neuroscience infant with the just-plain-bad-science bathwater.

The fundamental complaint is that “neuroscience inherited its vocabulary from philosophy and psychology.” But here, GB makes a generalization with which we disagree: the level of analysis which he objects to is essentially the level of folk-psychological terms. That is to say, GB disagrees with using terms such as volition, imagination, emotion, greed, etc., on the view that these are terms that are (the best of) what psychology and philosophy have to offer. But that is an unfair characterization both of contemporary psychology and cognitive science and of contemporary philosophy. In the terminology of the philosopher Wilfrid Sellars (Sellars, 1971), GB is criticizing brain–behavior research because it uses concepts from the “manifest image” perspective on the world when it should be building on the architecture of the “scientific image.” While there certainly exists research on the relationship between brain and behavior that is conceptually underspecified and folk psychological in its analytic approach, a substantial (and, happily, growing) body of inquiry is more subtle and sophisticated. (Brief side remark: it is at our peril that we simply dismiss concepts like imagination, greed, and so on. These common-sense concepts are used to provide plausible accounts of our behavior; we describe each other’s actions, beliefs, and desires rather successfully by deploying terms at this level of analysis. Discussing someone’s beliefs and behavior in terms of greed is straightforward and interpretable; trying to do the same thing by making reference to sodium channels or cortical columns or LTP is a category jump that is not plausible. Establishing principled, mechanistic relations between a broad category such as greed and its implementational infrastructure is extraordinarily difficult, obviously. But it is a strong “eliminativist” commitment to disregard psychological categories which are in fact successful at accounting at some abstract level for our cognition and behavior; cf. Fodor, 1987).

Where is there evidence of progress? Sticking to Sellars’s terminology, many of the psychological and philosophical terms used in the “manifest image,” say *imagination*, have been carefully analyzed and decomposed, and inserted into experiments which make the complex structure of these ideas visible and testable (adopting the “scientific image”). In perusing a recent compendium of the cognitive neurosciences (Poeppel et al., 2020), one can see many examples of this across the domains of perception, cognition, emotion, action, and so on. To provide one example, research that tries to relate language, a highly complex domain of human experience, to brain mechanisms takes advantage of highly granular, theoretically well motivated, and computationally explicit analyses that in aggregate constitute the domain of language. Importantly, the “parts list” that enters into such an analysis is supported by colossal amounts of data. Focusing, say, on the comprehension of spoken language, there is a tremendous amount of independent evidence that supports “representational primitives” such as distinctive feature, phoneme, syllable, morpheme, prosodic word, intonation contour, concatenation, binary set formation, etc. And to be totally clear: these elementary units of language comprehension are not hallucinated or ad hoc stipulations; large bodies of data from cross-linguistic comparisons, language acquisition, brain injury, computational linguistics, online processing, and even brain imaging provide compelling support that these units form the basis of online language comprehension in the human brain.

So, carrying on with this example, how are we to study the relationship between such elementary units and the brain? One way to conceptualize the problem is to think of it as an alignment problem between the set of primitives postulated by language research and the set of primitives postulated by neurobiology. Unless one assumes an (implicit or explicit) hierarchy of evidence, such that neurobiological data are more valid than other forms of data, then it stands to reason that one should try to find principled relations between the elements of the two domains. And indeed, there has been exciting progress in this regard. There are numerous experiments using sensitive and carefully interpreted neurophysiological results that show that representational primitives such as distinctive features or syllables have neurobiological implementations that can be methodically investigated (Mesgarani et al., 2014; Ding et al., 2016; Poeppel and Assaneo, 2020). The scientific inquiry then becomes a slightly different kind of challenge: on the one hand, we try to address a “*maps*” problem, i.e., establishing a functional anatomy of the form that has been extraordinarily successful in the neurosciences (retinotopy, somatotopy, the structure of the hippocampus, dual stream architecture underpinning language processing, etc.). On the other hand, we can and should tackle the more difficult “*mapping*” problem, i.e., the explicit alignment between the primitives of the cognitive domain, in this case language, and the primitives of neurobiology. The difficulties, promises, and consequences of this challenge have been spelled out (Poeppel and Embick, 2005; Poeppel, 2012; Embick and Poeppel, 2015). (The alignment problem, or the mapping problem, was particularly salient in the relationship between physics and chemistry at the end of the 19th and beginning of the 20th centuries. In that case, it was not a reduction that ended up being successful, but rather the integration or unification that required conceptual change on both parts. Physics had to make remarkable changes to its conceptual commitments to accommodate the new findings, and only thereby was there successful alignment between the phenomena investigated in the two disciplines.)

It goes without saying that solving the alignment problem between the primitives of the cognitive sciences and the primitives of neurobiology remains hugely difficult. As argued by Embick and Poeppel (2015), there are two problems to overcome, the granularity mismatch problem and the ontological incommensurability problem. The former is more of a practical constraint, the consequence of the fact that different fields look at phenomena at very different levels of resolution. The latter is a principled constraint, like in the case of chemistry and physics. Are the ontological commitments of neurobiology and psychology incommensurable, and is no alignment possible at all? In any case, the questions can be explicitly stated, turned into precise research hypotheses, and productively investigated. And this stance is compatible with both the inside-out approach advocated by GB (namely adopting the primitives of neuroscience to bootstrap the building blocks of psychology, insofar as this inductive step is possible) and the outside-in approach, for which GB takes as the paradigmatic exponent to be David Marr (Marr, 1982) and his levels-of-analysis heuristic.

We think that the prospects to understand the relation between brain and behavior are therefore less bad than GB does. While there is bad research in all areas of the sciences, the progress in the psychological and cognitive sciences, in particular by being careful about decomposing complex concepts, is leading to ever better hypotheses about how the brain underpins complex computation in the service of behavior. But addressing this challenge purely from the inside out, building on the implementational level of analysis and attempting to derive how behavior may or may not be organized, seems highly problematic. We remain convinced that substantive progress will only occur if both the evidence from behavior and from neurobiology are taken seriously in the same way; in fact, the relation may tilt the other way, with neuroscience needing behavior, as we have previously argued (Krakauer et al., 2017).

## Don’t Embrace the Epistemological Primacy of the Hardware

GB critiques the current approach to the relation between brain and behavior and, more broadly, what he calls the outside-in approach to neuroscience. What are the normative recommendations he derives? Here, we discuss some of his arguments in support of neuroscientific research that builds from “objective stuff” (the hardware of the brain, material stuff) up to the phenomena for which we use folk-psychological terms. (It should come as no surprise that we disagree with this epistemological conjecture.) Although we might not be able to build up from dendritic spine to “greed” (or volition, or imagination, etc.), it is implicit in the approach that GB endorses that we *could *build up from dendritic spine to “GREED,” which is loosely related to the concept of greed as used in common sense conception. GB outs himself here as a “radical implementationist” and inverse-Marrian (whose meta-scientific model to study complex systems he disavows).

### Motivating the implementation-first stance: problems

There is a type of historical example that has become popular among those advancing stridently reductionist views. (One can be an “implementationist” without being a radical reductionist, to be sure. We do not impute to GB that he is a radical reductionist, seeking to reduce all phenomena to quantum mechanics; just that he is an implementationist, such that an arbitrary level of implementation comes first.) Here is the argument: over time, our understanding evolves from explanations that use unobserved/unobservable entities to ones having observed, physical stuff at the center. The more unusual the initial explanation sounds, the better. “*When molecular genetics entered the scene, an essentially inside-out approach, 'élan vital' was eliminated from our vocabulary. After the discovery of DNA, this hypothetical expression was no longer needed to explain how a seed becomes a tree*” (Buzsáki, 2019). Such an example serves to support the view that it would have been more productive to just “start from the inside,” since this is where one ends up anyway. We wonder whether this might be too idealistic about the process of discovery and, in doing so, arrives at contentious conclusions. We draw attention to a possible oversight in the reductionist reading of the events: the supposedly absurd and ultimately demoted explanation actually embodies key necessary features of the explanatory entity, in this case, that there had to exist some internal driver of spontaneous morphogenesis. This interpretation of the scientific process has been discussed before, “*[…] it seems possible that at higher levels some important principles may be anticipated from behavioural evidence alone. The major principles of genetics were all inferred from external evidence long before the internal molecular structure of the gene was even seriously thought about*” (Dawkins, 1976). No matter how absurd, in hindsight, the interim explanations might sound, we should not let that cloud their real merits in the service of explanation and exploration, given the context of the knowledge base at the time. The epistemic processes leading to the discovery of mechanistic explanations are deeper and messier than the reductionist would have us believe.

### Advocating for implementation-first: more problems

In pitting outside-in against inside-out views, GB argues the latter are losing the popularity contest. “*[…] Neuroscientists less frequently take a well-defined physiological phenomenon (e.g., long-term potentiation) and examine the behavioral conditions (e.g., memory) that might match (correlate with) it.*” Here GB betrays a “radical implementationist” aspiration. Mainstream cognitive neuroscience is argued to follow a misguided strategy. “*This strategy is based on the assumption that the independent variable* [a cognitive construct] *represents a real, objectively existing entity*” (Buzsáki, 2020). At the core of this statement is an implicit hierarchy of evidence. If the evidence comes from neural measures, then the mechanism objectively exists. If it comes from cognitive measures, the matter remains to be settled, “*[…] there is little guarantee that these terms correspond to circumscribed brain mechanisms*” (Buzsáki, 2020). We readily grant that there is a mapping problem at the interface between cognitive science and neuroscience (Poeppel, 2012). Unless we pay attention to the potential granularity mismatch between our formal theories of cognition and neural implementation, we are not likely to learn much. But GB takes the claim one step further: cognitive science and neuroscience are not on an equal footing. We believe this goes too far. At every level of organization there may be new properties to explain. Therefore, understanding emergent behavior at higher levels requires research as fundamental as any other (Anderson, 1972). It would be remarkable if we could make meaningful upward predictions that span multiple levels of organization, from physiology to psychology. But success in reducing does not guarantee the same for constructing, and so this remains a problem for even the most mature disciplines. We are skeptical that neuroscience will prove to be the exception.

### Pitting implementation-guided against computational theory-guided approaches

We do not endorse the inside-out versus outside-in opposition. Nevertheless, as an attempt to clarify what is at stake, we recast the inherent tension between these epistemic procedures as that between *What is a mechanism for X?* versus *What is Y a mechanism for?* We exemplify them in turn. (1) *Here is an interesting capacity: the barn owl localizes sounds in the dark based only on auditory cues. What is the mechanism that explains how it solves this problem?* This structure to inquiry seems so intuitive that it hardly requires further scrutiny. We move on to its intended counterpart. (2) *Here are some interesting neural properties: neurons in the nucleus laminaris of the bird receive their excitatory inputs through axons of systematically varying lengths and with systematically varying interaural delays. What is this ensemble a mechanism for?* Now, there are several aspects of the latter that do not add up. For one, it is hard to imagine that scientists would even attempt so specific a characterization in the absence of either (1) a candidate phenomenon on the horizon, at the level of cognition and behavior, to be explained; or (2) an array of evidence from multiple levels of organization contextualizing the neural description that would justify the effort (Grothe, 2003, as well as Carr and Christensen-Dalsgaard, 2015, provide excellent accounts). One would be hard-pressed to come up with a consistent account of how researchers decided exactly what to look for. “*It is impossible to find nothing in the brain*” (Buzsáki, 2019). In fact, the only reason the second approach does not sound incoherent is that there is virtually always background information against which these kinds of “objectively real” neural properties are interpreted. Therefore, we argue that even if a bottom-up approach is conceivable and fruitful, it is substantially different from the idealized version. In other words, if the reader finds the second approach sounds problematic in comparison, it is only because this is rarely what happens.

### Background knowledge and the implementation sandwich

Background information, usually (although not exclusively) couched in the language of the primary explanandum (i.e., in this case psychology/behavior), precedes and supports the discovery of mechanistic explanations. Although this could be justified logically and historically, we conjecture that it corresponds to the underlying structure of neuroscientific reasoning as well. We illustrate this with the following example from GB’s 2019 book. “*First, there is the ‘good-enough’ brain. This is largely prewired and acts quickly via a minority of highly active and bursting neurons connected by fast-conducting axons and strong synapses into a network. The good-enough brain judges the events in the world in a fast and efficient way but is not particularly precise*” (Buzsáki, 2019). We dub this locution the “implementation sandwich.” A preview of the relevant cognitive level of organization, followed by the corresponding description of the neural substrate, followed by a more detailed cognitive-level description. This pattern is pervasive and diagnostic of the actual underlying reasoning that goes on, not just in the typical neuroscientist’s mind, but among the most avid advocates of the inside-out view. We find a similar pattern in the last chapter of GBs book, where Daniel Kahneman’s work in psychology is called on to contextualize and validate the outcomes of an inside-out program. Background information in the language of higher levels of organization holds the implementation sandwich together and guides the discovery process. We will revisit the role of background knowledge when we discuss real-world neuroscientific discovery as an abductive process in the last section.

### Implementationist aspirations: examples to ponder

Let us briefly consider a few cases that could, at least in principle, constitute examples where a thorough description of the hardware, the implementation level, could form the basis for inferring elements of psychological or behavioral phenomena. The connectome provides a case in point. In measuring everything from worms to undergraduates, the connectome has been welcomed as an important characterization of the neural substrate that will yield answers about foundational properties of behavior and cognition. Has the connectome actually generated the kinds of insight that one might expect and demand from this level of scientific effort? There is no debate that the thorough characterization of the wiring diagram of an animal’s nervous system can be immensely useful. But the direct inference from a wiring diagram to a function remains, at best, aspirational. This position is well expressed in a discussion of the (fully characterized) *Caenorhabditis elegans*: “*I think it’s fair to say…that our understanding of the worm has not been materially enhanced by having that connectome available to us. We don't have a comprehensive model of how the worm’s nervous system actually produces the behaviors. What we have is a sort of a bed on which we can build experiments, and many people have built many elegant experiments on that bed. But that connectome by itself has not explained anything*” (Movshon, 2012). In our view, this captures the issue quite well: this type of characterization of the hardware/wetware/implementation can help us formulate certain questions about function, but without those functional hypotheses clearly in our sights, it is unclear how to derive the computational infrastructure of the brain from even highly detailed structural characterizations.

The second example comes from research in which GB has played a central role: sharp wave ripples (SWR) in the hippocampus. In a masterful chapter in his recent book, GB describes the details of this physiological activity pattern (we are careful to avoid the term “response”) and its potential role. SWR are found in virtually every mammalian brain and play a role, descriptively, in the packaging of hippocampal spikes. What is their interpretation? The language that is used to describe a likely function is full of mentalist terminology: “*This peculiar and unique brain pattern is viewed today as a subconscious mechanism to explore the organism’s options, searching for stored items of the past in the disengaged brain* to *extrapolate and predict possible future outcomes. It embodies a brain mechanism that compresses the discrete concepts of past and future into a continuous stream*.” We agree that the research program is in part inside-out; GB first provides a detailed description of the physiological components of the SWR. But the interpretation is driven by hypotheses about behavioral or psychological functions, not, say, by temporal properties of the ripples. Again, this is an example of the “implementation sandwich,” with the psychological or behavioral or computational assumptions as implicit bookends for the analysis of the hardware.

Finally, let’s turn more explicitly to the computational approach. Suppose we have a computer chip about which we know essentially everything, since it is a designed artifact. We can now apply a suite of sophisticated measurement approaches to this chip. Will we be able to infer, on the basis of such measurements what the specific function of this chip is, let’s say in the context of some old game? The answer is, quite clearly, no. That is to say, even when we have a full characterization of the hardware, the inference to algorithm is either impossible or extraordinarily difficult or subject to chance (Jonas and Kording, 2017). From the perspective of current systems and computational neuroscience, the difficulty of going from computational characterization to algorithm to hardware (more or less the standard approach that GB calls outside-in) versus the difficult steps going from hardware to algorithm to computational theory has been critically addressed (Carandini, 2012).

## Don’t Forget: You Are an Implicit Philosopher

We turn, finally, to examine GB’s view of the (neuro)scientific process and the philosophical position this view entails. It is just as clear and concise in the recent book, “*A major advantage of the inside-out approach is that it is free from philosophical connotations.*” as it was 20 years ago: “*[Behavior terms] have been inherited from philosophy rather than emerging from objective investigation of the brain itself.*” First, we get our knee-jerk reaction out of the way and then move toward more subtle, controversial aspects that require careful consideration.

The view that one can do science unencumbered by philosophy we find untenable. Much ink has been spilled in challenging the notion that it is possible to free areas of scientific inquiry from philosophical assumptions (cf., in our own words, Krakauer et al., 2017); we will not elaborate here. The inside-out approach proposes to carve out “objectively interesting” neural anatomy/dynamics first, without the burden of cognitive science. “*I suggest that neuroscience, as any new discipline, should establish its own vocabulary based on brain mechanisms. It should start with the brain (independent variable) and define descriptors of behavior (dependent variables) that are free from philosophical connotations*” (Buzsáki, 2020). This idea is tempting, but what criteria should go into deciding what is interesting, helpful, useful? What are the decision and evaluation metrics? Whatever these are, they surely come from outside the system being studied. Needing these criteria to get the research off the ground, every scientist (tacitly or explicitly) turns to philosophical and psychological assumptions. The question shifts from whether or not any assumptions are made, to begin with, we think obviously yes, to whether one is prepared and willing to examine their implications.

This brings us to examine GB’s notion of how neuroscientific progress comes about. We believe that when confronted with actual practice, it reveals an idealized portrayal. “*[…] the only systematic strategy science possesses for the objective query of the simplest or most complex problem is hypothesis testing […] the key element in scientific progress is the formulation of a well-defined null hypothesis that can be rejected by diligent work.*” This picture naturally motivates GB to ask what the “null hypothesis for questions of the mind” could possibly be. But any puzzlement derived from this question is only a result of problematic premises regarding the process of science (on a tangentially related note, for a nuanced picture of scientific discovery that will likely take intuitions by surprise, see Devezer et al., 2019). We believe a less idealistic account than GB’s hypothetico-deductive picture (Popper, 1959), one that is more faithful to real-world practice, comes from considering what actually goes on in the lab, not merely what tends to be enacted in scientific articles. We must acknowledge that this tension does not go unnoticed, “*I tend to agree that many discoveries in neuroscience emerge from exploration […] Yet, when we analyze our data, interpret our results and present ideas to our peers, we describe our findings in the framework of hypothesis testing and support or reject them to communicate our conclusions to the community*” (Buzsáki, 2020). What is lacking is an account of how neuroscience makes progress that somehow reconciles these seemingly inconsistent practices.

### Abduction is a realistic approach

We find such an account in considering the goals of cognitive neuroscience within an abductive-theory-of-science framework. Abduction (Peirce, 1974), distinct from deductive and inductive inference, jointly captures the process by which a set of candidate explanations is generated from observations and background knowledge (sometimes termed abduction proper), and how the choice among them is justified (i.e., inference to the best explanation) (see Blokpoel et al., 2018). Cognitive neuroscientific theories aim to provide mechanistic explanations (cf. Bechtel, 2008). Importantly, the things to be explained are human cognitive faculties, and the types of mechanistic explanations inferred are those cast in terms of how the brain implements solutions to problems embodied by computational models (see Guest and Martin, 2020, for an analysis of the role of computational modeling in psychology). Through this lens, we might view the process of doing research in the field of cognitive neuroscience as the iterative abduction of certain kinds of mechanistic theories about human capacities. On this framework, a number of GB’s ideals, taken at face value, turn out to be incompatible with neuroscientific practice as we understand it. These are (1) that science is a series of rejections of null hypotheses; (2) that there is an implicit hierarchy of evidence, with neural evidence being promoted above cognitive science evidence; (3) that neural phenomena can be described entirely independently of background knowledge; and (4) that explanations “*emerge from objective investigation of the brain itself*.”

Rather than by proposing and rejecting strawman null hypotheses, progress in cognitive neuroscience, as we see it, derives from building (formal) theories of how capacities are instantiated, both algorithmically and implementationally. We should note that GB explicitly rejects the Marrian framework (Marr and Poggio, 1976; Marr, 1982), as it constitutes in GB’s terms a strongly outside-in stance: “*In contrast to the computational – algorithmic – implementation strategy, I argue throughout this book that understanding brain function should begin with brain mechanisms and explore how those mechanisms give rise to the performance we refer to as perception, action, emotion, and cognitive function*” (Buzsáki, 2019). We have suggested that there is an epistemological hierarchy in GB’s proposed strategy, wherein neural dynamics occupy a privileged spot. But on our view of the neuroscientific process, this means focusing on the wrong things. We have already covered the sense in which it is an eliminative neural implementationist stance, one that feels disconnected from practice. We highlight now a different aspect that does not fit: we are not interested only in explaining neural dynamics, this would be backwards. We are interested in explaining faculties in terms of theorized neural mechanisms that can make contact with observational/experimental data. We suspect that GB would agree with this direction of explanatory flow. Yet, the insistence on the reliability of neural effects would suggest otherwise. Here is why. Neural effects are only incidental to the particular realization of the capacities we are interested in explaining. As such they represent an opportunity to arbitrate between candidate likely (i.e., not strawman) explanations. This makes neural observables incredibly valuable epistemically, it does not, though, promote them to primary explananda (see Van Rooij and Baggio, 2020, for a discussion of similar curious substitutions). Formal theories in cognitive neuroscience are not constructed to account for the purportedly independent neural effects, however reliable, that we observe in the lab. Instead, they work out hypotheses about the realization, in both neural and cognitive algorithmic terms, of the capacity to be explained. A disproportionate focus on the objectivity and reliability of the ‘neural stuff’ of experiments distracts from a more crucial epistemic process: the exploration, not only of data, but of formal theoretical ideas.

Granted, theory building is an extremely complicated and challenging endeavor. It is natural to conclude that we are better off establishing the reliability of “*objective, real neural entities*” and working our way up (or in GB’s terms, out). But a fundamental limitation of the many scientific methods at our disposal is the difficulty in inferring theory from observations (Meehl, 1997). No amount of local inferences built on the ashes of null hypotheses can save us from this struggle (Newell, 1976). This is one of those problems that will just refuse to go away. Accounts ostensibly solving the problem of, in GB’s terms, “theory emergence” in a radical bottom-up way cannot omit exactly how they achieve such remarkable feats of bootstrapping (for an honest attempt with a humbling outcome, see Jonas and Kording, 2017).

Our position is not that the inside-out approach that GB advocates does not work, this would be factually incorrect. Rather, we argue that, insofar as it represents an epistemological and methodological alternative, we must necessarily be looking at a nuanced and realistic version of the approach: nuanced in the sense that it can avoid extreme views on the degree of independence neuroscience can achieve, and realistic in the sense that it can stay closer to how neuroscientists make progress in practice.

## Outro

Whether one agrees or disagrees, we think that it is great to think about the process of science in the context of philosophy. GB wrote a provocative and interesting paper 20 years ago, and he expanded many of those ideas in a fascinating and important book now. To build on a rich body of empirical work and think about how we can make progress on the deep and difficult questions of mind and brain is a really important goal. There is nothing wrong at all with sticking to careful quantitative descriptions of the “stuff that is the brain,” that is to focus on the implementational level of analysis. It is only when we begin to generate linking hypotheses between mind and brain that we come up against a series of methodological and philosophical challenges that merit consideration and debate. We pointed out a range of topics on which we disagree. But obviously, there is no single set of approaches that is correct, whatever that would mean (Feyerabend, 1975). In practice, there are likely a set of diverse approaches that reinforce each other’s presuppositions and biases, and in the absence of a compelling argument we should keep our options open.

When it comes to larger assumptions about the brain and its properties, we very much share the positions that GB is supporting. One hypothesis that we find particularly alluring is his notion of preformed brain dynamics. He argues convincingly against a tabula rasa approach and provides exciting clues how to think about the infrastructure or ‘operating system’ of the brain that comes for free. As he writes in the concluding chapter: “*If brain networks and dynamics are preformed, what advantages do they offer over the blank slate model? First and foremost, its preexisting ‘ideal forms’ provide the necessary balance to keep the brain’s dynamical landscape stable and robust against other competing needs, such as wide dynamic range, sensitivity, and plasticity*.” How the preexisting structures and the preformed dynamics underpin both externally elicited and endogenous experience remains to be understood. Whatever ends up being the most innovative and exciting and productive approach, few things could be more fascinating than trying to illuminate the complicated relation between the stuff of the brain and the stuff of the mind.

## Synthesis

Reviewing Editor: Christophe Bernard, INSERM & Institut de Neurosciences des Systèmes Decisions are customarily a result of the Reviewing Editor and the peer reviewers coming together and discussing their recommendations until a consensus is reached. When revisions are invited, a fact-based synthesis statement explaining their decision and outlining what is needed to prepare a revision will be listed below. The following reviewer(s) agreed to reveal their identity: NONE.

Nice opinion piece!
